# Risk Factors for Recurrent Urinary Tract Infections in Female Patients: A Systematic Review of Behavioral and Gynecologic Determinants

**DOI:** 10.7759/cureus.108846

**Published:** 2026-05-14

**Authors:** Sultan M Alhazmi, Mohammed A Khormi, Nura G Halawi, Rayan A Al-Abdulmughni, Mohammed A Al-Amri, Abdullah A AlAzzaz, Ahmed A Altalhi, Nouf M Adawi, Kayan M Alotaibi, Marie M Aljahdali, Talal K Aldawsari, Noor I Sherwani, Shaima K Khurmi, Nasser H Alowaimer, Fanan A Hakami

**Affiliations:** 1 College of Medicine, Jazan University, Jazan, SAU; 2 Family Medicine, Jazan University, Jazan, SAU; 3 College of Medicine, King Saud University, Riyadh, SAU; 4 College of Medicine, King Khalid University, Abha, SAU; 5 Faculty of Medicine, University of Tabuk, Tabuk, SAU; 6 Obstetrics and Gynecology, King Abdulaziz Medical City, Riyadh, SAU; 7 College of Medicine, King Faisal University, Al-Ahsa, SAU; 8 College of Medicine, University of Jeddah, Jeddah, SAU; 9 College of Medicine, Prince Sattam bin Abdulaziz University, Al-Kharj, SAU; 10 Faculty of Medicine, Jazan University, Jazan, SAU; 11 College of Medicine, Imam Mohammad Ibn Saud Islamic University, Riyadh, SAU

**Keywords:** behavioral factors, contraception, female, gynecologic factors, recurrent urinary tract infection, risk factors, sexual activity, spermicide, urinary tract infection

## Abstract

Recurrent urinary tract infections (rUTIs) are a common and burdensome condition among adult women, with a multifactorial etiology involving behavioral, gynecologic, host-related, and microbiological factors. Despite extensive research on individual risk factors, comprehensive evidence integrating these domains remains limited.

This systematic review was conducted in accordance with the Preferred Reporting Items for Systematic Reviews and Meta-Analyses (PRISMA) 2020 guidelines. A comprehensive search of PubMed, Scopus, Web of Science (WOS), and the Cochrane Library was performed from database inception to the final search date. Studies were included if they evaluated gynecologic and/or behavioral risk factors associated with UTI recurrence in adult women. Eligible study designs included observational studies and relevant clinical trials. Data extraction and methodological quality assessment were conducted using standardized tools, including the Mixed Methods Appraisal Tool (MMAT) and the modified Downs and Black checklist. Eleven studies met the inclusion criteria.

Sexual intercourse was identified as the most consistent and strongest behavioral risk factor, demonstrating a clear dose-response relationship with UTI recurrence. Spermicide-based contraception further increased risk, whereas hygiene practices showed inconsistent associations. Habitual urine holding emerged as a potential modifiable risk factor, while increased daily water intake was identified as the most robust protective factor, supported by evidence from randomized controlled trials. Gynecologic and host-related factors, including estrogen deficiency, urinary incontinence, pelvic organ prolapse, and comorbidities, were also associated with recurrence. A prior history of UTI and early-life susceptibility further contributed to increased risk. From a microbiological perspective, *Escherichia coli* predominated, with significant antimicrobial resistance observed among recurrent infections.

Overall, rUTIs in adult women are driven by a complex interplay of behavioral, gynecologic, host-related, and microbiological factors. Sexual activity and spermicide use represent the most consistent risk factors, whereas increased hydration appears to be the most effective non-pharmacological preventive measure. These findings support a multifactorial approach to prevention and highlight the need for individualized, evidence-based strategies to reduce recurrence and antimicrobial use.

## Introduction and background

Urinary tract infections (UTIs) are among the most common bacterial infections worldwide and represent a substantial burden on individual health and healthcare systems. Women are disproportionately affected due to anatomical and physiological factors, with approximately 50%-60% experiencing at least one UTI in their lifetime [1-4]. The clinical significance of UTIs is heightened by their tendency to recur. Recurrent urinary tract infection (rUTI) is typically defined as two or more episodes within six months or three or more episodes within 12 months, and it affects a substantial proportion of women following an initial infection, often leading to a chronic, relapsing course with negative impacts on quality of life, including physical discomfort, psychological stress, and disruption of daily and sexual activities [2,3].

A wide range of risk factors contributes to the development and recurrence of UTIs. In premenopausal women, key risk factors include frequent sexual intercourse, use of spermicides or barrier contraceptives, new sexual partners, and a personal or family history of UTI [5,6]. Sexual activity, in particular, has consistently been identified as the strongest behavioral risk factor, with a clear dose-response relationship between frequency of intercourse and infection risk. In postmenopausal women, risk factors shift toward host and gynecologic changes, such as estrogen deficiency, urinary incontinence, pelvic organ prolapse, and increased residual urine volume, all of which can facilitate bacterial colonization and persistence. Estrogen deficiency also alters the vaginal microbiota and reduces protective lactobacilli, weakening local defense mechanisms [7-11].

Behavioral and modifiable factors have been widely studied as potential targets for prevention. Hygiene practices show inconsistent associations with UTI risk, while voiding behaviors, such as delayed urination, may increase susceptibility in some individuals. Increased fluid intake has emerged as a protective factor, with evidence indicating that higher daily water consumption reduces recurrence rates [3,5,7]. Microbiologically, *Escherichia coli* remains the predominant causative organism, and rising antimicrobial resistance further complicates management. Despite extensive research on individual risk factors, existing literature remains fragmented, with limited comprehensive synthesis integrating behavioral, gynecologic, and microbiological contributors [1-5]. Therefore, this systematic review aims to identify and critically appraise the evidence on gynecologic and behavioral risk factors associated with rUTI in adult women, providing a more integrated understanding to inform prevention strategies and guide future research.

## Review

Methods

Literature Search Strategy

This systematic review was conducted in accordance with the Preferred Reporting Items for Systematic Reviews and Meta-Analyses (PRISMA) 2020 guidelines [12]. A comprehensive electronic search was performed in PubMed, Scopus, Web of Science (WOS), and the Cochrane Library from database inception to the final search date. The search strategy combined controlled vocabulary and free-text terms related to UTIs, recurrence, and gynecologic and behavioral risk factors in women. Keywords included variations of UTI, cystitis, recurrent infection, risk factors, and relevant exposures such as sexual activity, contraception, spermicide use, hygiene, voiding behavior, and hydration. The strategy was developed for PubMed and adapted for other databases. Searches were limited to human studies and original research articles, and non-relevant publication types were excluded (Tables 1-2).

**Table 1:** Search strategy used for literature retrieval This table presents the search strategy used to identify relevant studies in electronic databases, including PubMed, Scopus, Web of Science, and the Cochrane Library. Keywords and search terms were grouped by concept and combined using Boolean operators (AND, OR). The search strategy was adapted for each database using appropriate controlled vocabulary and syntax, where applicable.

Concept	Keywords / Search Terms
Condition	“urinary tract infection” OR “UTI” OR “cystitis”
Recurrence	“recurrent” OR “recurrence” OR “recurrent infection”
Population	“women” OR “female” OR “adult women” OR "adolescent"
Risk Factors	“risk factors” OR “behavior” OR “behavioral factors” OR “gynecologic” OR “sexual activity” OR “contraception” OR “spermicide” OR “hygiene” OR “voiding behavior” OR “hydration”

**Table 2:** PICO framework for study selection This table outlines the Population, Intervention/Exposure, Comparison, and Outcome (PICO) framework used to define the eligibility criteria for studies included in this systematic review. The framework guided study selection and ensured alignment with the review objective of identifying behavioral and gynecologic risk factors associated with recurrent urinary tract infections in adult women.

Component	Description
Population (P)	Adolescent and adult female patients with urinary tract infection (UTI) or recurrent urinary tract infection (rUTI), defined as ≥2 episodes in 6 months or ≥3 in 12 months
Intervention / Exposure (I)	Behavioral and gynecologic risk factors (e.g., sexual activity, spermicide use, hygiene practices, voiding behavior, hydration, estrogen status, urinary incontinence, pelvic organ prolapse)
Comparison (C)	Women without exposure to the specified risk factors or with lower exposure levels
Outcome (O)	Occurrence or recurrence of UTI (including frequency, incidence, time to recurrence, and/or culture-confirmed infection)

Eligibility Criteria

Eligibility criteria were defined a priori using a population-exposure-outcome framework. Studies were included if they involved adult or adolescent women with UTI or rUTI, defined as two or more episodes within six months or three or more within 12 months. Eligible studies assessed gynecologic, behavioral, or related risk factors and reported outcomes related to infection occurrence or recurrence. Observational studies and randomized controlled trials were included without restriction on geographic location or clinical setting. Studies were excluded if they did not involve adult women, did not assess relevant risk factors, lacked outcome data, or focused exclusively on microbiological or treatment interventions. Case reports, reviews, editorials, conference abstracts, and non-English publications were also excluded.

Study Selection

All retrieved records were imported into reference management software, and duplicates were removed. Study selection was conducted in two stages: initial screening of titles and abstracts, followed by full-text assessment of potentially eligible studies. Inclusion and exclusion criteria were applied consistently, and reasons for exclusion at the full-text stage were documented. The selection process was summarized using a PRISMA flow diagram.

Data Extraction and Quality Appraisal

Data extraction was conducted using a standardized, pre-piloted form. Extracted data included study design, setting, sample size, participant characteristics, risk factors assessed, outcome measures, and key findings. Methodological quality was evaluated using the Mixed Methods Appraisal Tool (MMAT) and the modified Downs and Black checklist [13,14]. These tools assess domains such as reporting quality, external validity, risk of bias, confounding, and statistical power. Quality assessment, informed interpretation of findings, and identification of potential sources of bias.

Results

Study Selection

**Figure 1:**
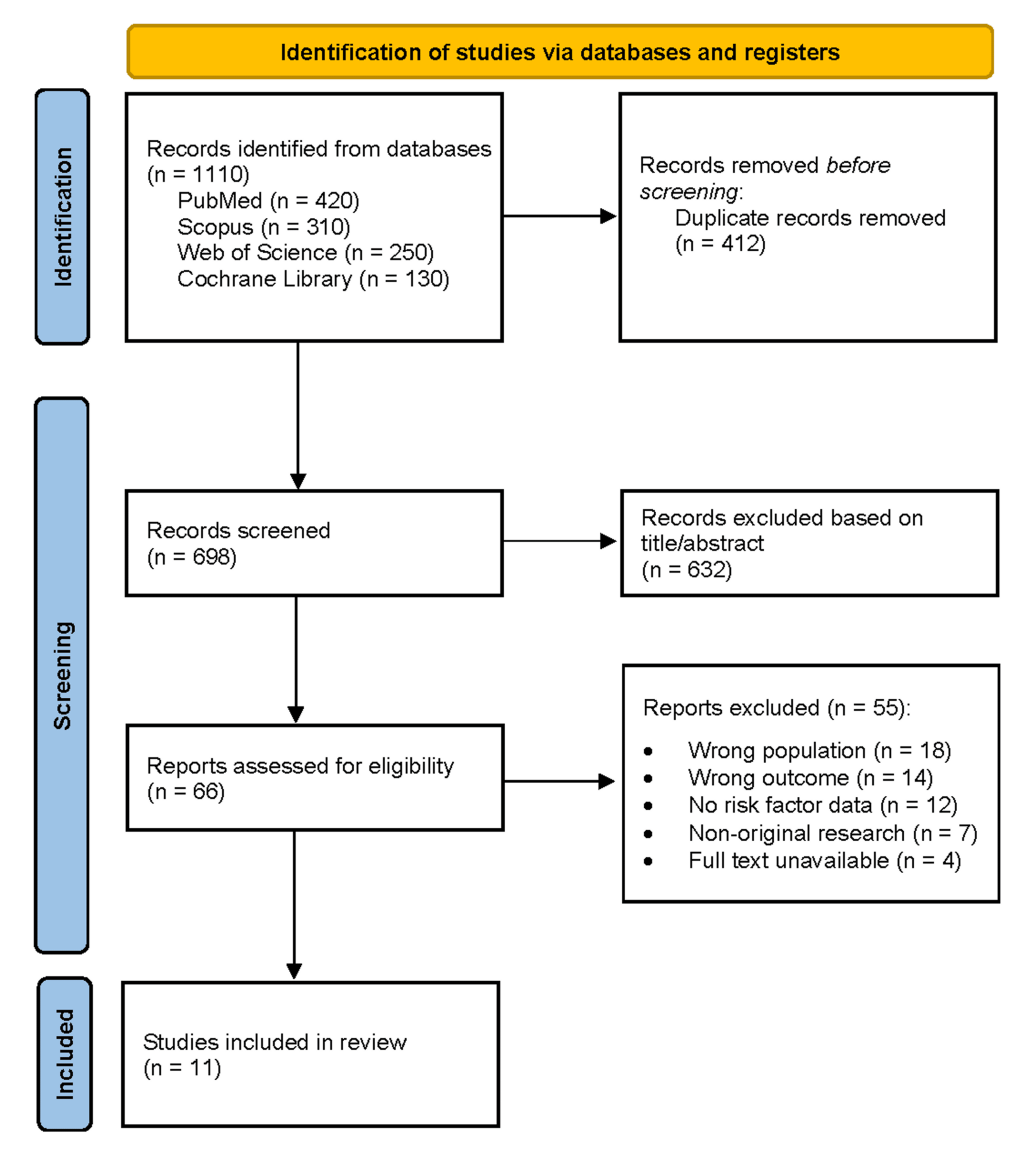
PRISMA flow diagram depicting the study selection process for the systematic review PRISMA (Preferred Reporting Items for Systematic Reviews and Meta-Analyses) flow diagram detailing the study selection process for this systematic review [12]. Following the identification of records through database searching and other sources, duplicates were removed, and the remaining records were screened. Full-text articles were assessed for eligibility, with exclusions documented along with reasons. Studies meeting the inclusion criteria were included in the final synthesis.

The database search identified 1,110 records, of which 698 remained after duplicate removal. Following title and abstract screening, 632 records were excluded. Sixty-six full-text articles were assessed for eligibility, and 55 were excluded due to inappropriate population, outcomes, study design, or unavailable full text. Eleven studies met the inclusion criteria and were included in the final qualitative synthesis (Figure 1).

Study Characteristics

The included studies varied in design, population, and focus. Several studies examined behavioral and sexual risk factors in premenopausal women, including exposures such as intercourse frequency, spermicide use, hygiene practices, voiding behavior, and prior UTI history [2,3,6-8]. Other studies focused on voiding behavior, hydration, or hygiene practices [4,9], while one randomized trial evaluated increased water intake in women with rUTI [5]. A smaller number of studies investigated host and biological mechanisms, including estrogen status, inflammation, and microbiological factors [10,11]. Outcomes included incident or recurrent infection, culture-confirmed infection, time to recurrence, and antimicrobial resistance patterns (Table 3).

**Table 3:** Characteristics and key findings of studies included in the systematic review on risk factors for rUTIs in adult women This table summarizes the main characteristics and findings of studies included in this systematic review investigating behavioral, gynecologic, and host-related risk factors for UTIs and rUTIs in adult women. Data presented include study country, design, population characteristics, sample size, assessed risk factors, statistically significant risk factors, outcome measures, and key findings. Risk factors were considered significant based on the original study reports. Outcomes include incident UTI, symptomatic UTI, or recurrent UTI, depending on study design and definition used. Study designs include cross-sectional studies, case-control studies, prospective cohort studies, retrospective cohort studies, and RCTs. Abbreviations: UTI, urinary tract infection; rUTI, recurrent urinary tract infection; USA, United States of America; OR, odds ratio; IL-6, interleukin-6; *E. coli*, *Escherichia coli*; RCT, randomized controlled trial.

Study ID	Country	Study Design	Population	Sample Size	Risk Factors Assessed	Significant Risk Factors	Outcomes Measured	Key Findings
Ackerson et al. [1]	USA	Retrospective cohort	Women with UTI	374171	Comorbidities, antibiotic exposure	Diabetes mellitus, antibiotic use, and age	rUTI incidence	rUTI rate 14.5%; associated with comorbidities
Scholes et al. [2]	USA	Case-control	Women (18-30 years)	482	Sexual, genetic, and contraceptive factors	Intercourse (OR 5.8-10.3), spermicide use, maternal history	rUTI	Strong behavioral and genetic risk profile identified
Hooton et al. [3]	USA	Prospective cohort	Young women	796	Sexual frequency, spermicide use	Intercourse, spermicide use, prior UTI	UTI	Demonstrated dose-response relationship
Jagtap et al. [4]	India	Cross-sectional	Women (reproductive age)	816	Holding urine, hygiene	Urine holding (OR 2.0), prior UTI	UTI prevalence	Behavioral and environmental risk factors identified
Hooton et al. [5]	USA	Randomized controlled trial	Premenopausal women	140	Hydration	Low fluid intake	rUTI frequency	Increased water intake reduced rUTI by ~50%
Moore et al. [6]	USA	Prospective cohort	Postmenopausal women	913	Timing of intercourse, diabetes mellitus	Intercourse (within 2 days), diabetes	Symptomatic UTI	Risk increased 3.4-fold within 2 days of intercourse
Ahmed et al. [7]	Saudi Arabia	Case-control	Women (18-40 years)	469	Sexual, hygiene, and urinary factors	Childhood UTI, wiping direction, intercourse, pelvic organ prolapse	rUTI	Multiple independent predictors (OR up to 6.8)
Seid et al. [8]	Ethiopia	Case-control	Women (15-24 years)	296	Sexual hygiene, voiding behaviors	Coitus, wiping, and delayed voiding	UTI	Strong associations reported (OR up to ~9.1)
Akaishi [9]	Japan	Cross-sectional	Adult women (female subgroup)	294	Wiping style, hygiene	Wiping (age-specific effect)	UTI occurrence	Significant association only in women aged 40-59 years
Meister et al. [10]	USA	Prospective cohort	Postmenopausal women	70	Estrogen status, inflammation	Estrogen deficiency	rUTI, IL-6 levels	Vaginal estrogen therapy reduced inflammation and rUTI
Czaja et al. [11]	USA	Prospective cohort	Premenopausal women	104	Sexual activity, colonization	Intercourse, *E. coli* colonization	rUTI	Infectious events precede recurrence by 2-3 days

Quality Assessment

Overall, the methodological quality of included studies was high based on the MMAT. Most studies used appropriate designs, representative samples, and valid measurement methods, with low risk of bias [2,3,5-8,11]. Some studies were rated as moderate quality due to limited adjustment for confounding variables, particularly cross-sectional analyses and large observational datasets [1,4,9,10]. These limitations suggest that certain findings should be interpreted with caution (Table 4).

**Table 4:** Methodological quality assessment of included studies evaluating risk factors for rUTIs in adult women using the MMAT Studies included in this systematic review were appraised using the MMAT (2018), with criteria applied according to study design, including randomized controlled trials, nonrandomized studies, and descriptive cross-sectional studies [13]. For each study, five design-specific methodological domains were assessed, including representativeness of the target population, appropriateness of outcome and exposure measurements, completeness of outcome data, control of confounding variables, and, where applicable, randomization procedures, blinding, and intervention adherence. Each criterion was rated as “Yes” or “No.” In accordance with MMAT guidance, no overall numerical score was calculated. Instead, methodological quality was summarized descriptively based on the criteria fulfilled and the primary methodological limitations identified for each study. The MMAT screening questions were applied prior to appraisal to confirm that the included studies addressed the research question and provided sufficient data for evaluation. Abbreviations: MMAT, Mixed Methods Appraisal Tool; rUTI, recurrent urinary tract infection; RCT, randomized controlled trial.

Study ID	Study Design	MMAT Criteria Assessed	Responses	Methodological Quality Summary
Ackerson et al. [1]	Cohort (Nonrandomized)	3.1 Representative of the target population? 3.2 Appropriate outcome/exposure measurements? 3.3 Complete outcome data? 3.4 Confounders accounted for? 3.5 Exposure/intervention administered as intended?	Y, Y, Y, N, Y	Met 4 of 5 MMAT criteria; confounding variables were not fully addressed.
Scholes et al. [2]	Case-control (Nonrandomized)	3.1 Representative of the target population? 3.2 Appropriate outcome/exposure measurements? 3.3 Complete outcome data? 3.4 Confounders accounted for? 3.5 Exposure/intervention administered as intended?	Y, Y, Y, Y, Y	Met all 5 MMAT criteria.
Hooton et al. [3]	Cohort (Nonrandomized)	3.1 Representative of the target population? 3.2 Appropriate outcome/exposure measurements? 3.3 Complete outcome data? 3.4 Confounders accounted for? 3.5 Exposure/intervention administered as intended?	Y, Y, Y, Y, Y	Met all 5 MMAT criteria.
Jagtap et al. [4]	Cross-sectional (Descriptive)	4.1 Relevant sampling strategy? 4.2 Representative sample? 4.3 Appropriate measurements? 4.4 Low risk of nonresponse bias? 4.5 Appropriate statistical analysis?	Y, Y, Y, N, Y	Met 4 of 5 MMAT criteria; risk of nonresponse bias was unclear or not adequately addressed.
Hooton et al. [5]	Randomized Controlled Trial (RCT)	2.1 Appropriate randomization? 2.2 Comparable groups at baseline? 2.3 Complete outcome data? 2.4 Blinded outcome assessment? 2.5 Participant adherence to intervention?	Y, Y, Y, Y, Y	Met all 5 MMAT criteria.
Moore et al. [6]	Cohort (Nonrandomized)	3.1 Representative of the target population? 3.2 Appropriate outcome/exposure measurements? 3.3 Complete outcome data? 3.4 Confounders accounted for? 3.5 Exposure/intervention administered as intended?	Y, Y, Y, Y, Y	Met all 5 MMAT criteria.
Ahmed et al. [7]	Case-control (Nonrandomized)	3.1 Representative of the target population? 3.2 Appropriate outcome/exposure measurements? 3.3 Complete outcome data? 3.4 Confounders accounted for? 3.5 Exposure/intervention administered as intended?	Y, Y, Y, Y, Y	Met all 5 MMAT criteria.
Seid et al. [8]	Case-control (Nonrandomized)	3.1 Representative of the target population? 3.2 Appropriate outcome/exposure measurements? 3.3 Complete outcome data? 3.4 Confounders accounted for? 3.5 Exposure/intervention administered as intended?	Y, Y, Y, Y, Y	Met all 5 MMAT criteria.
Akaishi [9]	Cross-sectional (Descriptive)	4.1 Relevant sampling strategy? 4.2 Representative sample? 4.3 Appropriate measurements? 4.4 Low risk of nonresponse bias? 4.5 Appropriate statistical analysis?	Y, Y, Y, N, Y	Met 4 of 5 MMAT criteria; risk of nonresponse bias was unclear or not adequately addressed.
Meister et al. [10]	Cohort (Nonrandomized)	3.1 Representative of the target population? 3.2 Appropriate outcome/exposure measurements? 3.3 Complete outcome data? 3.4 Confounders accounted for? 3.5 Exposure/intervention administered as intended?	Y, Y, Y, N, Y	Met 4 of 5 MMAT criteria; confounding variables were not fully addressed.
Czaja et al. [11]	Cohort (Nonrandomized)	3.1 Representative of the target population? 3.2 Appropriate outcome/exposure measurements? 3.3 Complete outcome data? 3.4 Confounders accounted for? 3.5 Exposure/intervention administered as intended?	Y, Y, Y, Y, Y	Met all 5 MMAT criteria.

Narrative Synthesis of Findings

Sexual activity was the most consistent and strongest behavioral risk factor for infection and rUTI across studies. A clear dose-response relationship was observed, with increasing frequency of intercourse associated with a higher risk of infection [2,3]. The association was consistent across age groups, although effect sizes were generally larger in younger women [6-8].

Spermicide use and certain contraceptive practices were associated with increased recurrence risk, and having a new sexual partner also contributed to higher risk [2]. These findings suggest that specific sexual practices may amplify susceptibility to infection.

Evidence on hygiene practices was inconsistent. Some studies reported increased risk with practices such as back-to-front wiping or douching [7,8], whereas others found no significant association [2]. Overall, the evidence for hygiene-related risk factors remains inconclusive.

Delayed voiding and habitual urine holding were associated with increased infection risk in some studies [4,8], although findings were not consistent across all studies. Postcoital voiding did not demonstrate a clear protective effect [3]. Increased fluid intake was the most consistent protective factor. A randomized trial showed that higher daily water intake significantly reduced recurrence and antimicrobial use [5], with supportive findings from observational studies [4,8].

Gynecologic and host-related factors, including urinary incontinence, pelvic organ prolapse, incomplete bladder emptying, and estrogen deficiency, were associated with increased recurrence risk [7,10]. Comorbidities and overall health status also contributed to susceptibility [1].

A history of prior infection was a consistent predictor of recurrence across studies [1,3,7,8]. Early-life infection and family history were also associated with increased risk, suggesting underlying susceptibility.

*E. coli* was the predominant pathogen across studies [3,4,6,8,11]. Antimicrobial resistance, including multidrug resistance, was frequently reported and associated with increased recurrence risk [1,8]. Evidence also supported the role of periurethral colonization and ascending infection in recurrence.

Discussion

Sexual activity was the most consistently reported risk factor across included studies, demonstrating a clear dose-response relationship with UTI recurrence. This association is biologically plausible, as intercourse facilitates the mechanical transfer of uropathogens, particularly *E. coli*, from the periurethral region into the urinary tract [3,14]. The temporal relationship observed in previous studies, with increased infection risk within 24-72 hours post-intercourse, further strengthens causality [6]. These findings are consistent with earlier landmark studies that identified sexual frequency as the primary predictor of UTI in young women [3,14].

The use of spermicides and barrier contraceptives was also associated with increased recurrence risk. Spermicides have been shown to disrupt the vaginal microbiota, particularly by reducing protective lactobacilli, thereby promoting colonization by uropathogens [6,8]. This mechanism is well-established and supports clinical recommendations advising against spermicide use in women with recurrent infections [9].

In contrast, evidence regarding hygiene practices remains inconsistent. While some studies suggest that wiping direction or genital hygiene may influence infection risk, others have found no significant association [2,7,8]. This inconsistency suggests that hygiene-related factors may be less influential than traditionally assumed or may be confounded by other behavioral and host-related variables. Similar conclusions have been reported in prior reviews [10].

Voiding behavior, particularly delayed urination, was identified as a potential modifiable risk factor. Urinary stasis may facilitate bacterial growth and increase the likelihood of infection [4]. However, findings were inconsistent across studies, indicating that the effect size may vary depending on individual physiology and environmental factors.

In contrast, increased fluid intake demonstrated the most consistent protective effect. A randomized controlled trial showed that increasing daily water intake significantly reduced rUTI recurrence rates by approximately 50% [5]. This effect is likely mediated by increased urine output, which promotes bacterial clearance and reduces colonization of the urinary tract [11]. These findings are supported by additional observational and interventional studies emphasizing hydration as a key preventive strategy [11,12].

A history of prior UTI was consistently identified as a strong predictor of recurrence, suggesting underlying host susceptibility or persistent bacterial reservoirs [1,3]. Emerging evidence indicates that intracellular bacterial communities may contribute to recurrent infections by evading host immune responses and antibiotic therapy [7].

*E. coli *remains the predominant causative organism in both initial and rUTIs [3,11,14]. Its virulence is attributed to factors such as adhesins, biofilm formation, and intracellular persistence, which enhance its ability to colonize and evade host defenses [1,5,8]. A concerning finding across studies is the increasing prevalence of antimicrobial resistance, including multidrug-resistant strains [1,8]. This trend complicates treatment and highlights the importance of non-antibiotic preventive strategies. Antimicrobial stewardship is therefore critical in managing rUTIs, as repeated antibiotic exposure may further drive resistance [3].

Limitations

Several limitations should be acknowledged. Heterogeneity in study design, populations, exposure definitions, and outcome measures precluded meta-analysis and limited the precision of the findings. The relatively small number of included studies (n = 11) may not capture the full scope of evidence. Many studies relied on self-reported data, introducing potential recall bias and measurement error, particularly for behavioral exposures. Some cross-sectional studies did not adequately control for confounding, limiting causal inference. The focus on gynecologic and behavioral factors excluded other relevant contributors, such as pharmacologic and microbiological influences. Additionally, variation in geographic and healthcare contexts may affect the generalizability of the findings.

## Conclusions

rUTI in adult women is a multifactorial condition shaped by the interaction of behavioral, gynecologic, host, and microbiological factors. Sexual activity and spermicide use appear to be the most consistent risk factors, while increased fluid intake represents a practical and effective non-pharmacological preventive measure. Other contributors, including voiding habits, hormonal status, urinary tract function, comorbidities, and prior infection history, likely interact to influence individual susceptibility to recurrence.

These findings support a comprehensive and individualized approach to prevention that incorporates behavioral modification, optimization of gynecologic health, and judicious use of antimicrobials. Further high-quality research with standardized methodologies is needed to clarify the relative contribution of different risk factors and to inform more targeted and sustainable prevention strategies.
